# Case report: a step-by-step body contouring approach in a case of young patient with CLOVES syndrome

**DOI:** 10.1080/23320885.2023.2290532

**Published:** 2023-12-08

**Authors:** Stefano Vaccari, Beniamino Bortoli, Camilla Maria Ester Bonzi, Arianna Balza, Edoardo Caimi, Riccardo Di Giuli, Flavio Bucci, Stefania Andreoletti, Valeriano Vinci, Francesco Klinger

**Affiliations:** a Department of Medical Biotechnology and Translational Medicine BIOMETRA, Plastic Surgery Unit, BIOMETRA, Humanitas Clinical and Research Hospital, Reconstructive and Aesthetic Plastic Surgery School, University of Milan, Milan, Italy; b Department of Biomedical Sciences, Humanitas University, Milan, Italy; c Humanitas Clinical and Research Center-IRCCS, Milan, Italy; d Department of Health Sciences, Ospedale San Paolo, University of Milan, Milan, Italy

**Keywords:** CLOVES, macrosomy, lipoaspiration, dermolipectomy

## Abstract

CLOVES syndrome is a rare overgrowth disorder caused by gene mutations. This case study describes a 28-year-old woman with CLOVES syndrome who underwent multiple surgeries to achieve a positive outcome while preserving lymphovascular structures. The report underscores the importance of a multidisciplinary approach and tailored surgical interventions for managing CLOVES.

## Introduction

CLOVES syndrome is a rare, nonheritable sporadic segmental mosaic overgrowth syndrome. The number of worldwide published cases of CLOVES syndrome is between 130 and 200 cases thus far.

The term CLOVES is an acronym that denotes congenital lipomatous overgrowth, vascular malformations, epidermal nevi, and spinal/skeletal anomalies.

With this paper, we report a case of CLOVES syndrome in Italy, highlighting its characteristics and our approach in the management of lipomatous and epidermal overgrowths that affect this patient [1,2].

CLOVES syndrome is currently included in the ‘*PIK3CA*-related overgrowth spectrum’ (PROS): a heterogeneous collection of rare disorders sharing the same pathogenetic mechanism, involving postzygotic somatic gain-of-function mutations of the *PIK3CA* gene on chromosome 3q26.32. This provokes a disruption in the downstream regulation of the *PI3K/AKT/mTOR* signal transduction pathway, ultimately leading to uncontrolled growth of cutaneous, vascular, adipose, neural, and musculoskeletal tissues in a mosaic pattern, reflecting the body parts affected by the mutation [3].

Lipomatous masses that characterize CLOVES are present from birth and are usually accompanied by capillary malformations appearing as port-wine stains in the truncal region and on the extremities, with or without skeletal overgrowth.

The diagnosis of CLOVES syndrome is based on clinical examination, specifically concerning the vascular, cutaneous, spinal, truncal and limbs anomalies [4]. No histopathological evaluation is required, however, to assess the differential diagnosis with Proteus syndrome, genetic testing should be performed [5].

Although no clear therapeutic guidelines have been established, treatment must have a multidisciplinary approach where surgeries and lipoaspirations may play an important role for the management of the lipomatous overgrowths leading to decreased quality of life.

## Methods

**Figure 1.**
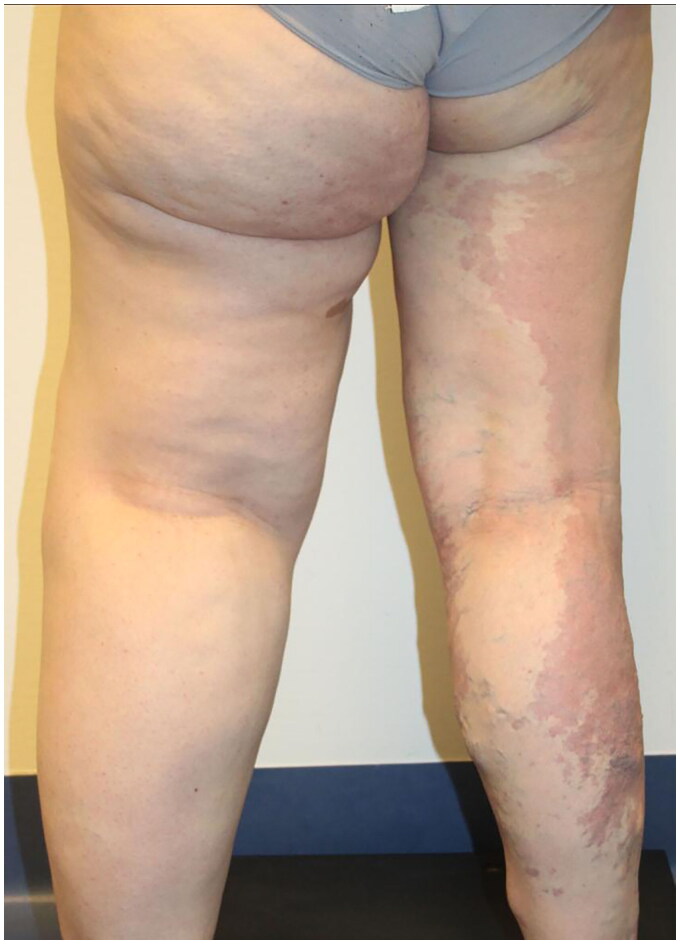
Standing posterior view of the patient before any surgical interventions.

We report the case of a 28-year-old Caucasian woman, who came to the attention of the Plastic Surgery department due to the presence of left leg macrosomy with excess of cutaneous and subcutaneous soft tissues, resulting from venous-lymphatic malformation (Figure 1).

At the age of 7, she underwent a safenectomy of the right leg, at the age of 9–10, she had multiple varicectomies of the right leg. At the age of 13, she underwent an epiphysiodesis of the left leg to surgically correct the leg length discrepancy. A more recent varicectomy of the right leg was performed at the age of 27.

**Figure 2.**
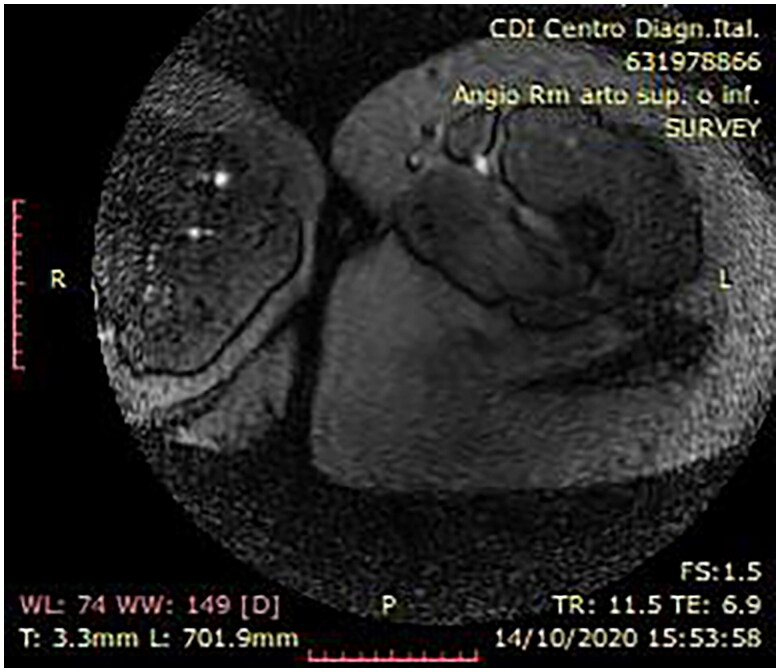
Preoperative MRI.

A radiological work up of the patient was performed through chest x-ray and contrast-enhanced MRI. The former, by means of the antero-posterior project, reveled a slight spinal scoliosis. The latter specifically revealing fatty replacement of the anterior and medial compartment of the left thigh, at T1-weighted sequences (Figure 2). At T1-weighted enhanced sequences, diffused vascular malformations were identified in the left thigh.

Following a multidisciplinary consultation, the patient was submitted to surgery.

In June 2021, we performed a suction-assisted lipectomy (approximately 2000 mL of lipoaspirate) of the internal and external thigh, trocanteric and subgluteal region of the left leg.

The left gluteal region was still notably afflicted by dermal and adipose excess, resulting in a significant functional constraint and a high degree of asymmetry with respect to the contralateral gluteal region.

In November 2021, the patient underwent another aspiration lipectomy (approximately 1600 mL of lipoaspirate from the anterior, lateral and medial left thigh) combined with resection of wide lozenge of skin and subcutaneous tissue from the left gluteal region.

The patient returned to our institution in June 2022 referring the persistence of functional limitations caused by the remaining presence of dermal and adipose excess in the left leg. Also, a hypertrophic erythematous scar was reported in the subgluteal fold, resulting from previous dermo-adipose resection.

**Figure 3.**
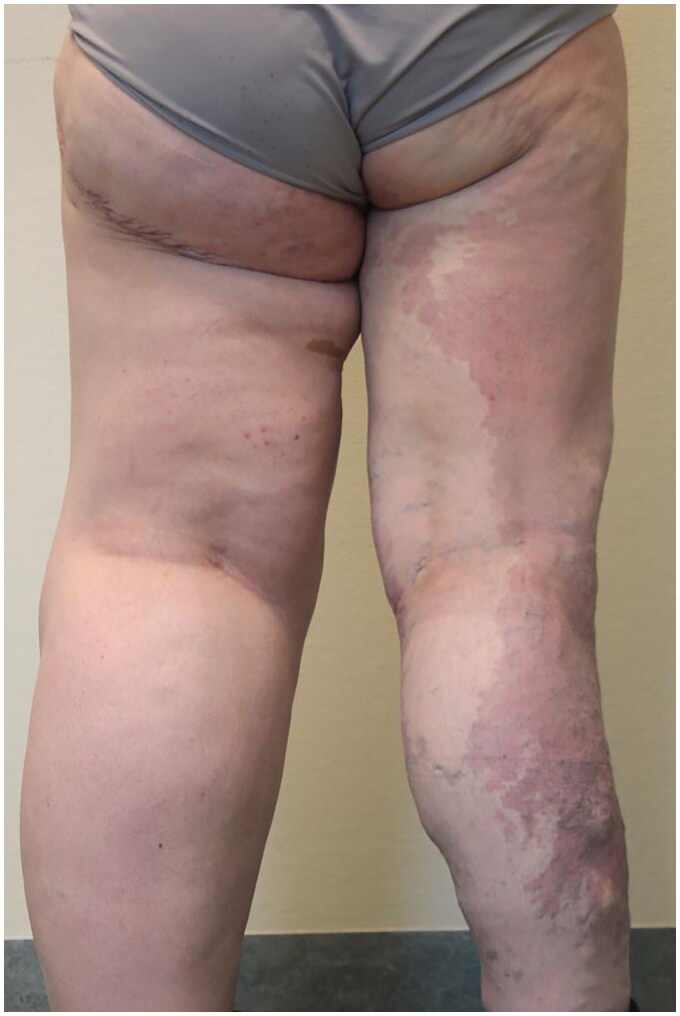
Standing posterior view of the patient after liposuction and tissue removal surgeries.

In June 2022, she underwent a further resection of a wide lozenge of skin and subcutaneous tissue of the left medial thigh via surgical asportation combined with lipoaspiration of the anterior and medial left thigh and of the genial region (approximately 1000 mL of lipoaspirate). (Figure 3)

After a thorough multidisciplinary consultation, the patient underwent a series of surgical procedures. These interventions were necessitated by the surplus adipose tissue, which created surgical complexities due to the need to consider the patient’s vascular and skeletal anomalies, thereby heightening the risk of bleeding. Notably, there were no instances of recurrence in this patient. The successive surgeries were carefully planned to safeguard vascular integrity, significantly mitigating the risk of bleeding. This approach also contributed to enhancing the patient’s functional outcomes.

Following the last surgery, the patient was followed up every month for six months via outpatient care, reporting subjective amelioration of her thigh circumference. During the last checkup, she reported to be satisfied with the morpho-functional result obtained and expressed her willingness to undergo further surgical procedures, if deemed appropriate.

## Discussion

For decades, diagnosis and management of overgrowth syndromes has been a challenge.

In addition to tailored medical treatment and a proper multidisciplinary approach, appropriate perioperative management is crucial for these patients, as large excisions may lead to damage to lymphatic components, significant blood transfusions and prolonged hospital stay [6].

Prior to 2004, the surgical techniques implemented in the management of lipodermatous overgrowths involved removal of tissues by full thickness excision. Le Louarn then introduced a new approach by describing liposuction of the suprafascial and subfascial component in a cutaneous resection area, performing cutaneous resection just under the dermis to preserve connective tissue containing not only the blood vessels but also the lymphatic vessels, thereby reducing the risk of postoperative seroma and lymphoedema [7].

Contrarily to what previously described, the most experienced author recommended performing the resection first and the liposuction afterwards in combined procedures; this was done to calibrate the amount of liposuction to be performed based on the condition of the flaps and their vitality after removal.

Extensive suprafascial liposuction has to be performed up to the top of the thigh as to avoid injury to the saphenous vein and the lymphatic network. Indeed, a recent anatomical study showed that the lymphatic vessels are present not only in subfascial fat, but also in the deep layer of superficial fat [8].

Surgical interventions were performed on the supra-fascial plane, in order to preserve the vascular, nervous and lymphatic integrity as much as possible [9,10].

Beyond surgical interventions, it is crucial to recognize the emerging significance of pharmacological therapies, especially targeted treatments, in the management of these patients, potentially assuming a pivotal role in the future. Notably, Rapamycin (sirolimus), an mTOR inhibitor, has demonstrated its efficacy in various instances involving patients with CLOVES syndrome, leading to improvements in adipose overgrowth, vascular irregularities, and overall quality of life [11,12]. Remarkably, regarding innovative target therapy, Pagliazzi et al. [13] documented a case where Alpelisib, a selective inhibitor of the catalytic subunit of PI3K, resulted in a reduction of lipomatous overgrowth. Apart from medical and surgical interventions, laser therapy has also shown effectiveness in addressing vascular malformations. However, it is crucial to adhere to specific indications for its application [14].

Overall, overgrowth syndromes represent a challenge for the plastic surgeon, who must tailor the available techniques for each specific case in order to put in place the best treatment to improve patient’s quality of life. A multi-step approach allows for detailed customization of the final result by giving the possibility to plan any subsequent surgical approach on a case-by-case basis, taking into consideration the patient’s achievements as well as his clinical condition.

We used our consolidated experience in the field of cosmetic surgery by applying the techniques of groin-cruroplasty, glutheoplasty and liposuction in order to achieve the best morpho-functional result for our young patient, minimizing possible complications, further manifesting the philosophy that the boundary between reconstructive and aesthetic surgery is extremely thin.

## Conclusion

As it emerged from this case-report, the management of CLOVES syndrome relies on a multidisciplinary approach, in which the clinical examination, radiological work up and surgery are fundamental. Therefore, from this case, we want to highlight the chief importance of a multi-step lymphovascular-spearing surgical approach thought the utilization of different techniques such as lipectomies and skin resections, precisely tailored on the needs of the patient.
